# Clinicopathological Features and Therapeutic Outcomes of Head and Neck Sarcomas: A 14-Year Retrospective Study

**DOI:** 10.7759/cureus.95070

**Published:** 2025-10-21

**Authors:** Chiraz C Halwani, Wafa Atrous, Sana Ferchichi, Sonia Esseghaier, Moncef Aloui, Ghassen Chebbi

**Affiliations:** 1 Otorhinolaryngology, Military Hospital of Tunis, Tunis, TUN; 2 Otorhinolaryngology, Mohamed Taher Maamouri Hospital, Nabeul, TUN; 3 Radiology, Military Hospital of Tunis, Tunis, TUN; 4 Maxillofacial Surgery, Military Hospital of Tunis, Tunis, TUN

**Keywords:** head and neck, head and neck sarcoma, management, radiotherapy, sarcoma, surgery

## Abstract

Head and neck sarcomas are rare malignant tumors with a wide histological diversity. Their diagnosis and management remain challenging because of their complex anatomical location and aggressive behavior. The objective of this study was to analyze the epidemiological, clinical, and paraclinical characteristics of head and neck sarcomas over 14 years in a tertiary military hospital, with a focus on survival, recurrence, and treatment response. A retrospective descriptive study was conducted in the ENT Surgery Department of the Military Hospital of Tunis from January 2010 to December 2023. Nineteen patients diagnosed with head and neck sarcomas were included. Data regarding demographics, clinical presentation, imaging, histology, treatment, and outcomes were analyzed. All patients underwent radiological evaluation to determine locoregional and distant tumor extension. Histological diagnosis was obtained by biopsy in 10 cases and from surgical specimens in nine. The study included 10 males and nine females, aged two to 76 years (mean age = 43 years). The most frequent histological subtypes were synovial sarcoma and embryonal rhabdomyosarcoma. Surgery was the main treatment in nine patients, often combined with adjuvant radiotherapy or chemotherapy. Reconstructive surgery using a frontal flap was performed in one case of nasal angiosarcoma. Two patients were treated with concomitant radio-chemotherapy, four received exclusive radiotherapy, and one patient received only chemotherapy. Distant metastases were identified in two cases. The mean follow-up was 24 months (range: two months to five years). Local recurrence occurred in two patients, and four patients experienced disease-related mortality. Head and neck sarcomas require complex and multidisciplinary management. Surgery remains the cornerstone of treatment when feasible, but therapeutic strategies must be individualized based on tumor location, histological type, and extension. Optimizing outcomes depends on early diagnosis and tailored, case-specific multimodal approaches.

## Introduction

Sarcomas are rare malignant tumors of mesenchymal origin, accounting for less than 1% of all adult cancers but up to 15% of malignancies in the pediatric population [1,2]. Head and neck sarcomas represent only 1-2% of all head and neck malignancies [3], making them an uncommon and challenging entity for diagnosis and treatment. These tumors exhibit significant histological heterogeneity, encompassing a wide range of soft tissue and bone sarcomas. Their anatomical location, often in proximity to critical neurovascular and functional structures, further complicates management and may compromise surgical margins [4].

Although wide surgical excision remains the cornerstone of treatment, optimal care frequently requires a multidisciplinary approach involving radiation therapy and/or chemotherapy, tailored to histological subtype, tumor stage, and patient-specific factors [5]. The prognosis remains guarded, with local recurrence and distant metastases being common, particularly in high-grade sarcomas.

The objective of this study was to analyze the epidemiological, clinical, and paraclinical characteristics of head and neck sarcomas over 14 years in a tertiary military hospital, with a focus on survival, recurrence, and treatment response.

## Materials and methods

A retrospective descriptive study was conducted in the ENT Surgery Department at the Military Hospital of Tunis over 14 years, from January 2010 to December 2023. Nineteen patients diagnosed with head and neck sarcoma were included. Patients were excluded if their medical records were incomplete or if they were followed up outside the hospital, and relevant data were missing from the hospital records.

Data were collected from patient records and included demographic characteristics, clinical presentation, imaging studies, histopathological findings, therapeutic strategies, and follow-up outcomes. All cases had a histologically confirmed diagnosis of sarcoma involving the cervico-facial region. Locoregional and distant extension assessments were systematically performed using contrast-enhanced cervico-facial CT scans. Additional imaging, such as magnetic resonance imaging, was requested when deeper anatomical areas or skull base invasion were suspected. Whole-body CT was performed to evaluate distant metastases. Histological confirmation was obtained either by biopsy or from the operative specimen following tumor excision. Treatment decisions were made during multidisciplinary tumor board meetings, including ENT, oncology, radiology, and radiation therapy specialists. Decisions based on resectability, histology-specific chemosensitivity, radiotherapy indications following international guidelines, and patients were managed according to tumor location, histological type, and extent of disease. Follow-up data included clinical evolution, recurrence, progression, and survival.

The study was conducted in accordance with institutional and national research ethics guidelines. Ethical approval was not required for retrospective analyses of anonymized data in our department.

## Results

Nineteen patients were treated for cervico-facial sarcoma during the study period. The mean age at diagnosis was 32 years (range: 2-76 years), with a slight male predominance (sex ratio M/F = 1.11). No familial history of cancer was reported. Two patients had a prior history of head and neck malignancy: one had undergone surgery followed by radioactive iodine therapy for papillary thyroid carcinoma, and another had received exclusive radiotherapy for nasopharyngeal undifferentiated carcinoma. No occupational exposure to carcinogens or genetic predisposition factors was identified. Only one patient was an active smoker.

The most common clinical presentations were cervical or facial swelling, bilateral nasal obstruction, and, less frequently, dysphagia. Physical examination findings varied depending on tumor location, with facial swelling being the most commonly observed sign. In all patients, a comprehensive general examination was performed, revealing no evidence of distant metastases or synchronous malignancies.

Imaging workup included CT scans in all patients, which provided detailed information on bone involvement and tumor extension. MRI was additionally performed in seven cases to better assess soft tissue invasion, particularly in rhabdomyosarcomas and parapharyngeal tumors. In one patient, cervical MRI demonstrated a hypopharyngeal tumor with laryngeal extension and bulky metastatic lymphadenopathy. Two patients presented with locally advanced rhabdomyosarcomas.

Histological diagnosis was established by biopsy in eight cases and by analysis of surgical specimens in eleven cases.

**Figure 1:**
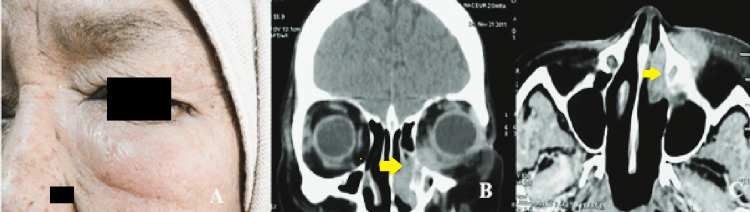
Case of a 62-year-old woman with a lacrimal sac low-grade fibromyxoid sarcoma. (A) Clinical presentation showing internal canthal swelling. (B, C) Coronal and axial CT scan views demonstrating a tumoral mass occupying the medial canthus with extension into the medial periorbital fat (yellow arrow). Histopathological examination confirmed a rhabdomyosarcoma of the lacrimal sac.

One patient, a 62-year-old female, was diagnosed with a low-grade fibromyxoid sarcoma of the lacrimal sac. She presented with an internal canthal swelling. Coronal and axial CT scans demonstrated a tumoral mass occupying the medial canthus with extension into the medial periorbital fat (Figure 1).

**Figure 2:**
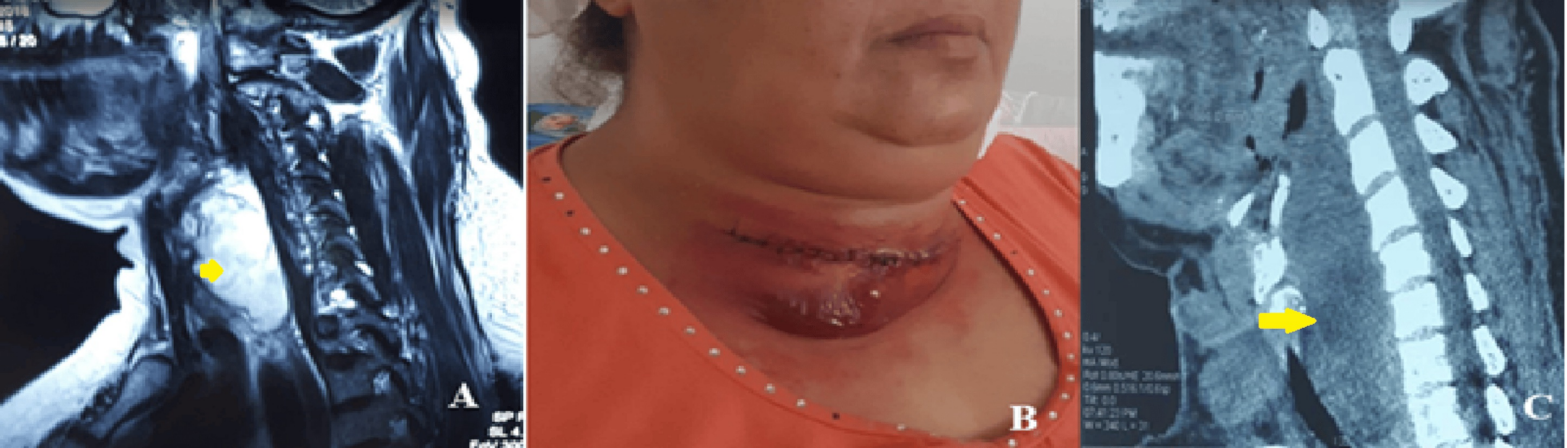
Case of a 42-year-old woman with a cervical synovial sarcoma. (A) Sagittal MRI showing a well-defined retroesophageal mass (yellow arrow) in a patient with a history of papillary thyroid carcinoma, initially suggestive of metastatic recurrence. (B) Rapid tumor progression after surgical exploration and biopsy. (C) Sagittal postoperative CT scan showing a poorly defined, infiltrative tumor (yellow arrow).

Another case of synovial sarcoma involving the cervical smooth muscles was diagnosed in a 42-year-old female. The patient had a history of papillary thyroid carcinoma and presented with dysphagia. Sagittal MRI revealed a well-defined retroesophageal mass, initially suggestive of metastatic recurrence (Figure 2).

**Figure 3:**
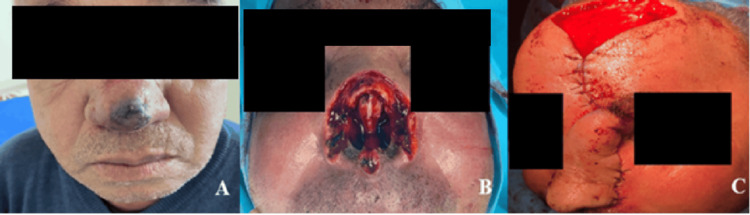
Case of a 70-year-old patient with a nasal angiosarcoma. (A) Clinical presentation of the tumor. (B) Intraoperative view after tumor resection. (C) Reconstruction of the defect using a frontal flap.

Surgical excision with curative intent was achieved in nine patients. One 70-year-old patient with nasal angiosarcoma underwent nasal reconstruction using a paralateral nasal flap (Figure 3), while another required selective neck dissection.

Adjuvant radiotherapy was administered in four cases due to histological aggressiveness or positive/close surgical margins. Exclusive chemotherapy was used for rhabdomyosarcoma cases. Two patients with advanced, non-operable disease received palliative chemotherapy, while one patient was treated with adjuvant chemotherapy. In a case of rapidly progressive leiomyosarcoma, concurrent radiochemotherapy was performed owing to the impossibility of surgical revision.

All patients underwent systematic metastatic staging with thoraco-abdomino-pelvic imaging. Two cases of metastatic disease were identified: pulmonary metastases in a patient with submandibular carcinosarcoma and bone metastases in a patient with parapharyngeal synovial sarcoma.

The mean follow-up duration was 24 months (range: two months to five years). Disease progression occurred in two patients, and local recurrence was observed in one case. Patients with poor local control had no remaining therapeutic options. In cases of obstructive tumors, tracheostomy and gastrostomy were performed to secure the airway and maintain adequate nutrition.

The management strategies, histological types, and patient outcomes are summarized in Table 1.

**Table 1:** Summary of histopathological types, tumor locations, surgical and adjuvant treatments, and clinical outcomes.

Histological type	Localization	Surgical procedure	Adjuvant treatment	Follow-up
Low-grade fibromyxoid sarcoma (N = 1)	Lacrimal sac	Dacryocystectomy	Postoperative radiotherapy	Good local control
Synovial sarcoma (N = 4)	Smooth neck muscle	Cervicotomy (positive margins)	Postoperative chemotherapy	Uncontrolled tumor progression
	Piriform sinus	Total hypopharyngolaryngectomy	Adjuvant radio-chemotherapy	Good local control, no recurrence
	Sinonasal	—	Palliative chemotherapy	Bone and pulmonary metastases
	Parapharyngeal	Lateral pharyngotomy	Postoperative radiotherapy	Bone metastases
Angiosarcoma (N = 2)	Nose	Rhinectomy + frontal flap reconstruction	Postoperative radiotherapy	Good local control, recurrence
	Cervical	Surgical resection (negative margins)	—	Good local control, recurrence
Leiomyosarcoma (N = 2)	Maxillary sinus	Biopsy	Neoadjuvant chemotherapy	Tumor progression, no remaining options
	Maxillary sinus	Endonasal resection	—	Excellent local control, no recurrence
Chondrosarcoma (N = 1)	Mandible	Left hemimandibulectomy	Radio-chemotherapy	Good local control, no recurrence
Embryonal rhabdomyosarcoma (N = 4)	Maxillary sinus	Endonasal maxillectomy	Adjuvant chemotherapy	Good local control, no recurrence
	External auditory canal	Biopsy	Adjuvant chemotherapy	Tumor progression
Botryoid rhabdomyosarcoma (N = 1)	Infratemporal fossa	Surgical resection	Adjuvant chemotherapy	Good local control
Carcinosarcoma (N = 2)	Submandibular gland	Submandibulectomy + neck dissection	Postoperative radiotherapy	Pulmonary metastases/good local control
Liposarcoma (N = 1)	Parotid	Total parotidectomy	Radio-chemotherapy	Good local control
Undifferentiated small round cell sarcoma (N = 1)	Hypopharynx	—	Chemotherapy	Poor local control

## Discussion

The epidemiological data in this study are consistent with published literature on head and neck sarcomas. Although limited by its retrospective single-center design, small cohort size, and incomplete chemotherapy details, which preclude detailed analysis of prognostic factors or statistical correlations, the study provides valuable insights into this rare and complex group of cervicofacial sarcomas, with detailed diagnostic and clinical follow-up data.

Soft tissue sarcomas have an estimated incidence of 3-4.5 cases per 100,000 individuals annually and represent approximately 1% of adult malignancies [1,2]. Most series report fewer than 50 cases, with the largest cohort including around 100 patients [2]. In children, soft tissue sarcomas account for about 35% of all sarcomas [3]. These tumors are classified into soft tissue sarcomas and bone/cartilage sarcomas, with an approximate distribution of 80% and 20%, respectively [4].

About 70% to 80% of head and neck sarcomas occur in adults, predominantly angiosarcoma, pleomorphic sarcoma, Kaposi’s sarcoma, and fibrosarcoma, whereas 20% to 30% affect children, mainly as osteosarcomas, rhabdomyosarcomas, and Ewing sarcomas [6]. Approximately 80% of tumors originate in soft tissues, and most cases arise sporadically without identifiable genetic predisposition [7]. Some studies suggest environmental and immunological factors, such as trauma and chronic infections, may contribute to sarcoma development [8].

Clinically, patients typically present with symptoms related to local tissue invasion. Tumors involving the skull base can cause diplopia, exophthalmos, facial pain, and headaches. Rhinosinus involvement often leads to nasal obstruction or epistaxis, while laryngeal tumors may result in dysphonia or dyspnea [6]. The classic diagnostic triad consists of a palpable mass, pain, and functional impairment [9].

International guidelines recommend multidisciplinary discussion before biopsy. Core needle biopsy under radiological guidance, with a sensitivity exceeding 90%, is preferred over open biopsy to reduce the risk of tumor seeding and morbidity [10]. Although the World Health Organization (WHO) recognizes over 50 sarcoma subtypes [10], current staging primarily relies on tumor grade, size, depth, and presence of metastases [2]. Imaging plays a critical role in diagnosis, staging, biopsy guidance, and assessment of resectability. CT excels at detecting bone involvement and calcifications and is advantageous for small tumors near air-filled sinuses or the skull base, where magnetic resonance imaging (MRI) artifacts may occur [10]. Positron emission tomography (PET)/CT offers high sensitivity and specificity for sarcomas, aiding initial staging, treatment response evaluation, and prognosis. PET/MRI further improves soft tissue and functional assessment for preoperative planning and neoadjuvant therapy guidance.

Head and neck sarcomas should be treated in a reference center, with multidisciplinary staff following national network guidelines [11]. The primary treatment goal is local control and prevention of distant metastases, which account for most sarcoma-related deaths [9,12]. Surgery with wide margins, followed by radiotherapy, remains the standard approach for resectable tumors, while perioperative chemotherapy is reserved for chemosensitive subtypes [8]. Achieving oncologic margins in the head and neck is challenging due to proximity to vital structures and concerns regarding postoperative function and aesthetics [5]. The decision to perform a biopsy or surgical excision for the histological diagnosis in our series was made based on the clinical presentation, the radiological assessment of tumor extensions and anatomical relationships, as well as the potential resectability of the lesion. Radiological imaging, particularly CT and MRI, played a crucial role in evaluating local invasion and guiding the most appropriate therapeutic approach. Lymph node dissection is not routinely performed but is indicated for clinically or radiologically suspicious nodes or for sarcomas with a known propensity for lymphatic spread, such as embryonal rhabdomyosarcoma and epithelioid sarcoma [8]. In cervicofacial sarcomas, lymph node involvement ranges from 3% to 10% [2]; in our series, neck dissection was performed in a case of submandibular carcinosarcoma.

Free flap reconstruction has been reported in the literature, with rates ranging from 7% to 24.3% in various series [4]. In our series, it was performed in one patient with a nasal angiosarcoma, using a frontal flap for reconstruction. Reconstruction choices depend heavily on surgical expertise and team preferences. Radiotherapy has a limited role except in Ewing’s sarcoma or in cases of residual disease when surgery is incomplete or unfeasible. Indications for postoperative radiotherapy include high-grade tumors, positive margins, tumors larger than 5 cm, and recurrences [2]. Chemotherapy is mainly adjuvant and often combined with surgery or radiotherapy, with variable response depending on sarcoma subtype [1]. Targeted therapies offer promising options for advanced or metastatic disease.

Head and neck sarcomas generally have a poorer prognosis compared to sarcomas at other sites due to anatomical complexity limiting wide excision [13]. Exceptions with better outcomes include orbital and non-parameningeal rhabdomyosarcomas [5]. Poor prognostic factors include tumor size greater than 5 cm, high histological grade, extension to skin, bone, or neurovascular structures, and positive surgical margins, all associated with higher recurrence and mortality rates [8,9]. For bone sarcomas, tumors larger than 6 cm, patient age over 60 years, osteoblastic histology, initial non-surgical treatment, and positive margins are unfavorable prognostic indicators [9]. The quality of surgical resection remains a major determinant of local control and survival, explaining the relatively better prognosis for mandibular sarcomas, where wide excision is more feasible [9]. In our series, the patient with mandibular sarcoma had a favorable outcome, whereas the patient with maxillary sarcoma had a poorer prognosis, likely due to prior irradiation.

Given the small sample size and limited follow-up, survival outcomes reported here should be interpreted cautiously and primarily as descriptive observations. Pediatric cases were few, and separate subgroup analysis was not feasible, so conclusions mainly reflect the adult cohort. Median overall survival in our cohort was 18 months, consistent with reported five-year survival rates ranging from 50% to 70%, depending on histological subtype [2].

## Conclusions

Cervico-facial sarcomas are rare and diverse tumors requiring multidisciplinary management. Early diagnosis and complete surgical excision combined with appropriate adjuvant therapy improve local control and patient outcomes. Histological subtype and tumor location significantly influence prognosis and treatment options. Despite advances, cases with poor local control remain challenging, highlighting the need for novel therapeutic approaches. Further studies with larger cohorts are essential to understand these tumors better and optimize management strategies.
